# Topological Models for Open-Knotted Protein Chains Using the Concepts of Knotoids and Bonded Knotoids

**DOI:** 10.3390/polym9090444

**Published:** 2017-09-13

**Authors:** Dimos Goundaroulis, Neslihan Gügümcü, Sofia Lambropoulou, Julien Dorier, Andrzej Stasiak, Louis Kauffman

**Affiliations:** 1 Center for Integrative Genomics, University of Lausanne, 1015 Lausanne, Switzerland; dimoklis.gkountaroulis@unil.ch (D.G.); julien.dorier@unil.ch (J.D.); Andrzej.Stasiak@unil.ch (A.S.); 2 SIB Swiss Institute of Bioinformatics, 1015 Lausanne, Switzerland; 3 Department of Mathematics, National Technical University of Athens, Zografou Campus, 15780 Athens, Greece; nesli@central.ntua.gr (N.G.); sofia@math.ntua.gr (S.L.); 4 Vital-IT, SIB Swiss Institute of Bioinformatics, 1015 Lausanne, Switzerland; 5 Department of Mathematics, Statistics and Computer Science, University of Illinois at Chicago, Chicago, IL 60607-7045, USA

**Keywords:** protein knots, knot theory, topology, knotoids

## Abstract

In this paper we introduce a method that offers a detailed overview of the entanglement of an open protein chain. Further, we present a purely topological model for classifying open protein chains by also taking into account any bridge involving the backbone. To this end, we implemented the concepts of planar knotoids and bonded knotoids. We show that the planar knotoids technique provides more refined information regarding the knottedness of a protein when compared to established methods in the literature. Moreover, we demonstrate that our topological model for bonded proteins is robust enough to distinguish all types of lassos in proteins.

## 1. Introduction

Interest in knots in protein structures dates back almost 25 years [1]. The existence of knots in a protein backbone results in kinetic and folding difficulties. As such, one may expect a bias against the formation of knots during evolution, but this is not the case. It has been conjectured that knots may provide structural advantages such as protection against degradation, thermal stabilization, or formation of regions with an increased number of contacting amino acids, which is a favorable environment for catalytic sites [2,3]. These factors, together with the belief that knowledge of the folded state of a protein may provide insight into the folding pathways that the backbone has followed makes the understanding of the topology of a protein chain very important.

Traditional methods of studying the entanglement of a protein implemented mathematical tools borrowed from knot theory that required that the backbone be a closed loop. In recent works, proteins were analyzed in terms of their global topology as open chains using the concepts of knotoids [4] and virtual knots [5,6]. Both methods produce similar results, yet the knotoids approach appears to be more sensitive in detecting pre-knot formations in the protein backbone [4].

Until now there has been no purely topological way to study the entanglement of an open protein chain that takes also into account the possible bridges (e.g., disulphide bridges) that may appear in the protein’s conformation. However, there are geometric methods that consider structures containing a wide range of bridge types (cysteine bridges, C-N interactions, C-O interactions, etc.) and covalent cofactor bonds [7,8,9,10,11,12]. These studies concluded that knots and links in proteins may appear by carefully selecting closed paths found in parts of the polypeptide chain, cofactors and disulphide bridges. On the other hand, studies on RNA folding [13,14] produced a topological method that effectively studied the folding of such biomolecules by interpreting folding nodes as rigid vertices and then substituting tangles at the bonded sites.

In this paper, we first present a refinement of the methods of [4] using the concept of planar knotoids and we show that it produces an even more detailed overview of the topology of an open protein chain. Then, we adapt the methods found in [13] to the case of proteins with disulphide bonds and we establish a robust, purely topological model that is able to distinguish such proteins in terms of their topology. Finally, we apply our model to the study of lassos which are entanglement motifs that arise in the conformations of proteins with cystine bridges and have been recently introduced by Niemyska et al. in [9] and studied further in terms of their classification using computational tools [10,15].

## 2. Knotoids

**Figure 1:**
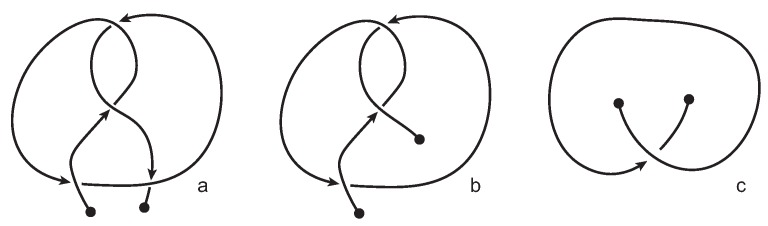
Various types of knotoids. A knot-type knotoid (**a**); a proper knotoid (**b**); and a non-trivial planar knotoid (**c**).

Knotoids are open-ended diagrams whose endpoints can be in different regions of the diagram, generalizing in this way the notion of a long knot, or a 1-1 tangle, and thus providing solid foundations for the definition of open knots (see Figure 1). Knotoids were first introduced by Turaev [16] and were further studied by Neslihan Gügümcü and Louis Kauffman [17].

In formal mathematical terminology, a *knotoid diagram* is defined as a generic immersion of the unit interval in an oriented surface Σ that appears as a curve in the plane which crosses itself with different tangent directions at each crossing point. The crossing points are endowed with the extra information of over-/under-passing arcs. Moreover, they are finite and isolated so that we never see more than two local segments of the curve crossing one another. The endpoints of the interval are carried through the generic immersion to two distinct points in Σ that are called the *tail* and the *head* of the knotoid diagram, or just *endpoints* of the knotoid diagram, and they are distinct from all of the double points [16,17]. A knotoid diagram is by convention oriented from tail to head. The knotoid notion can be extended to the notion of a *multi-knotoid*, which is a union of one long segment and a finite number of loops. Such an extension can be seen as an image of the unit interval and several unit circles $S^{1}$ under a generic immersion.

**Figure 2:**
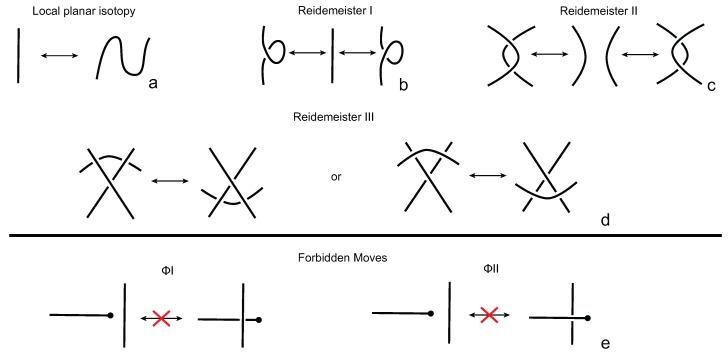
The Reidemeister moves (**a**–**d**) and the forbidden moves (**e**) on a knotoid diagram.

Two knotoid diagrams are called *isotopic* if one can be deformed to the other by a finite number of elementary moves on the diagrams called *Reidemeister moves* (see Figure 2a–c) that are performed away from the endpoints, together with local isotopy moves of the diagrams that result from isotopy deformations of the surface Σ such as stretching, shrinking and bending. Note that the sliding of an endpoint over or under other parts of a knotoid diagram is forbidden in the theory of knotoids. The forbidden moves are illustrated in Figure 2d. The Reidemeister moves together with local planar isotopy moves generate an equivalence relation on the set of knotoid diagrams in Σ and the corresponding equivalence classes are called *knotoids* [16]. Depending now on whether the endpoints of the diagram lie in the same or different local region of Σ, we distinguish knotoids into two categories: *knot-type knotoids* and *proper* knotoids (see Figure 1a,b). In a knot-type knotoid diagram, if one decides to close the diagram with an arc, the newly introduced arc may not create any additional crossings to the diagram.

**Figure 3:**
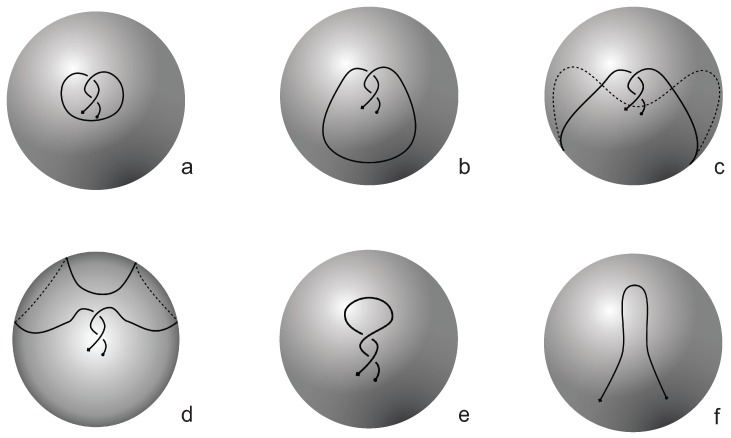
The planar knotoid $k2.3^{p}$ is trivial when considered in $S^{2}$. We start by placing $k2.3^{p}$ in $S^{2}$ (**a**). Then, the lower arc is stretched (**b**) and pulled towards and past the south pole, around the back (**c**) and finally is brought up again (**d**). The application of two Reidemeister I moves completes the proof (**e,f**). The dotted lines indicate parts of the diagram that are on the back side of the sphere.

In [16,17] knotoids are mostly studied on the 2-sphere $S^{2}$. In this paper, we consider knotoids in the two-dimensional (2D)space $R^{2}$. More specifically, *planar knotoids* are defined as equivalence classes of knotoid diagrams in the plane $R^{2}$ (see Figure 1c). Planar knotoids provide a more refined way to classify knotoids since there are examples of knotoids that are non-trivial on the plane but become trivial when they are considered in $S^{2}$. This is because we can always take an arc of the knotoid diagram in $S^{2}$, push it towards a pole of the sphere, across it and all the way around the surface of $S^{2}$ (see Figure 3) [16,17]. If we consider the same knotoid as in the previous example on a plane, we will see that we can deform and twist the knotoid but we will never have the freedom to move arcs in a way that will unknot the curve without violating the forbidden moves. Note from Figure 1c and Figure 3 that knot-type knotoids in the plane can be quite different from knot-type knotoids in the sphere.

As explained in [17], a knotoid diagram gives rise to a multitude of embedded open curves in the three-dimensional (3D) space in the following way. We consider the plane of the diagram is the horizontal plane in 3D-space. One pushes the overpasses of the diagram slightly into the upper half-space and the underpasses into the lower half-space while keeping the endpoints attached on two distinct lines passing through the endpoints, and are perpendicular to the plane that the diagram lies in. In this way one obtains a series of embedded open curves in $R^{3}$, all of which can be deformed to one another with respect to those infinite lines (see Video S1).

A three-dimensional curve on the other hand corresponds to many different knotoid diagrams since different projection directions may yield different and non-equivalent diagrams (see Video S2). Thus, the knottedness of an embedded curve in the 3D space is a probability distribution over all of its possible projections. Sampling the distribution allows one to approximate the dominant knotoid, which is the knotoid that has the highest probability of appearance.

## 3. Results

### 3.1. Analyzing Open Protein Chains Using Knotoids

Proteins appear in various, quite often very complicated conformations and, as such, the study of their topology has proven to be a challenge. In order to determine the entanglement type of a protein, one usually considers its backbone as an open polygonal curve and then simplifies it by applying an algorithm that preserves the underlying topology. Probably the most well-known technique in the literature is the triangle elimination or KMT (Koniaris-Muthukumar-Taylor) algorithm [18,19,20]. Many suggestions have been made on how to close an open 3D curve and they fall into two large categories [1,3,19,21,22,23,24,25]. First are the single closure techniques, such as the direct closure [19] and the out of the center of mass closure [3] techniques, where the chain is closed by a single arc that connects the endpoints. These methods are computationally fast but depending on the particular closure recipe the same protein may end up forming different knots. The second category comprises probabilistic methods such as the uniform closure technique [22,24,25], where the chain is first placed inside a large enough sphere (usually a radius of twice the length of the chain will be enough) and a simplification algorithm is applied. Each point of the sphere is now a possible closure point of the open chain. The closure is achieved by picking a point and then extending two rays, one from each endpoint of the chain, towards the chosen point and connecting them. The knot type, as in the case of knotoids that was discussed above, is a probability distribution. Such methods are less biased but they are more computationally intensive as the knot type of each closure has to be computed. Both categories have the disadvantage of altering the geometry of the studied object.

If one now chooses to study the entanglement of the protein backbone using the concept of knotoids, then the procedure is similar to the case of the uniform closure. Once again, the protein chain lies into a large enough sphere, but in this case each point of the sphere corresponds to a projection direction on a surface that lies outside the sphere. When the projection direction is determined, the two infinite lines are introduced and a simplification algorithm is applied on the chain in a way that the infinite lines are never crossed. The results then can be summarized on a map that identifies regions on a sphere and each distinct region corresponds to the projection directions in spherical coordinates that produce the same knotoid type. Moreover, each distinct region is color coded according to the knotoid type it carries. We shall call such a map the projection globe of a protein.

**Figure 4:**
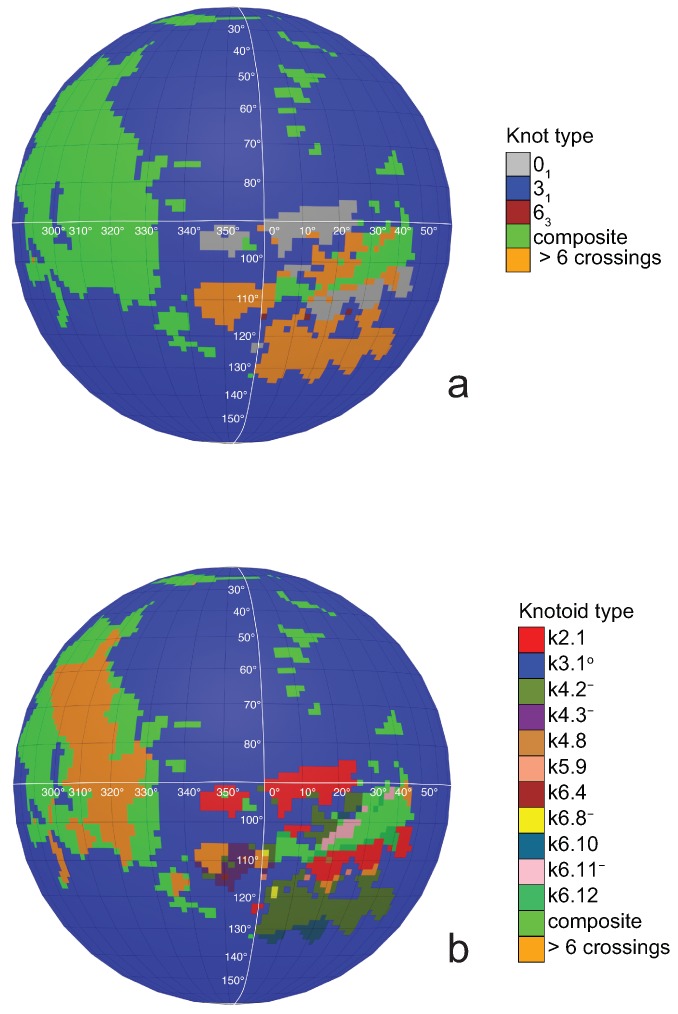

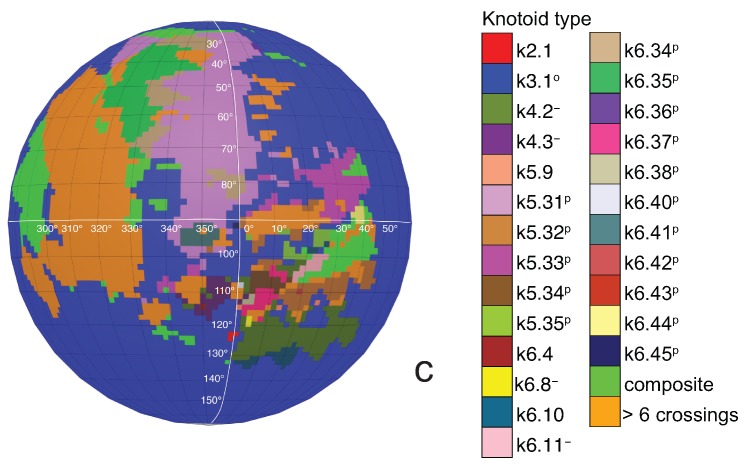
The projection globes for (**a**) the uniform closure technique; (**b**) the spherical knotoids technique; and (**c**) the planar knotoids technique.

**Figure 5:**
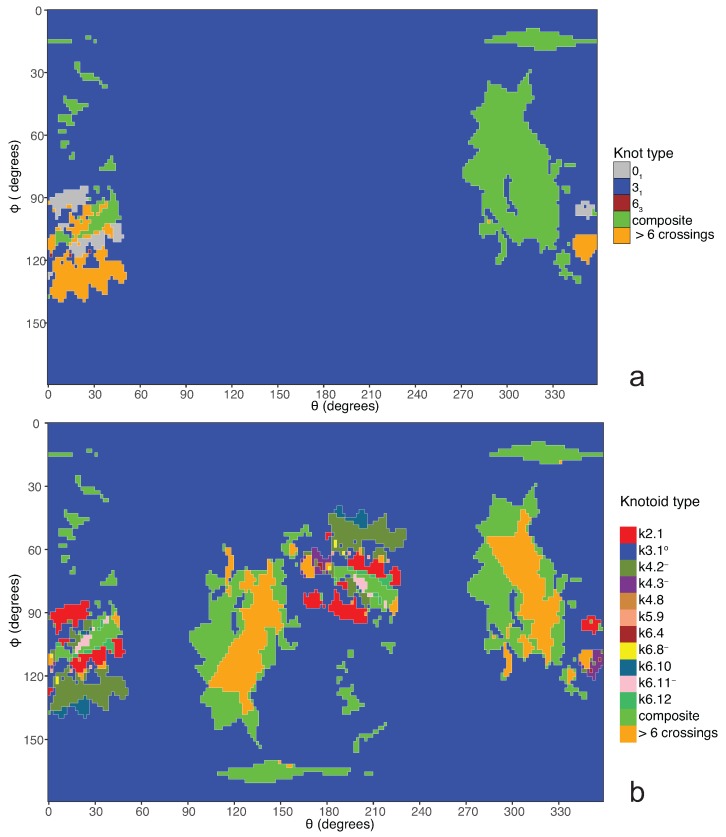

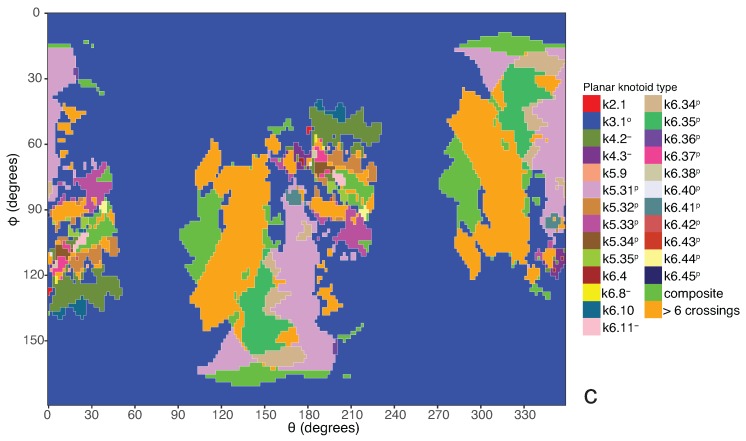
The projection maps for (**a**) the uniform closure technique; (**b**) the spherical knotoids technique; and (**c**) the planar knotoids technique.

Coming back to the discussion in Section 2 and considering the differences between planar and spherical knotoids, one can further refine the projection map of an open protein chain by refraining from pushing arcs around the surface of the sphere and considering projections of the chain on a plane instead. This will allow projections that were previously detected as unknotted to emerge as non-trivial planar knotoids. In this paper, we apply this approach to the protein with Protein Database (PDB) entry 3KZN (*N*-acetyl-l-ornithine) [26]. This protein is known to form a trefoil knot or a $k3.1^{\circ }$ knot-type knotoid. Recall that knot-type knotoids have both endpoints in the same region of the diagram and if one decides to close the diagram with an arc, the newly introduced arc may not create any additional crossings to the diagram. In our notation, a knotoid is represented by kX.Y, where *X* is the number of crossings of the knotoid diagram in question and *Y* corresponds to the position of the knotoid in our table among knotoids with the same number of crossings. Moreover, an exponent ${}^{\circ }$ indicates a knot-type knotoid, an exponent ${}^{p}$ a planar knotoid, and an exponent ${}^{-}$ a knotoid with its crossings inverted. Comparing now the projection globe obtained from the planar knotoids approach to the one derived from the spherical knotoids approach, as well as to the one that is derived from the uniform closure technique, we can see that new regions are gradually emerging as we move from knots to spherical knotoids and then to planar knotoids (see Figure 4). The reason behind this is that the number of classes of planar knotoids is larger than the number of classes of spherical knotoids, as discussed in Section 2 and shown in Figure 3. The diagrams in Figure 3 show a configuration that will simplify if the arc surrounding the endpoints could be swung all the way around a two- dimensional sphere. This cannot be done in the plane, and thus the diagram represents a knotoid that is not trivial in the plane but is trivial on the sphere. Figure 5 shows equirectangular projections of each projection globe shown in Figure 4. From the above we can conclude that analyzing open protein chains as planar knotoids reveals more details of their topology.

### 3.2. A Topological Model for Bonded Open Protein Chains

Motivated by the ideas in [13,14], in this section we introduce a purely topological model for analyzing the topology of bonded open protein chains in terms of planar (multi-) knotoids.

**Figure 6:**
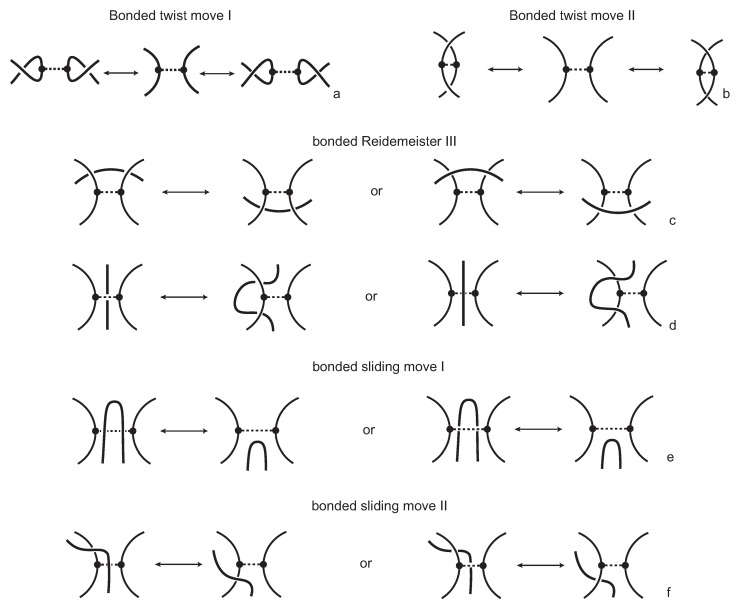
The bonded moves.

A bonding site of a protein chain consists of two local strands of the chain and a bonding arc with its ends based on these strands, as illustrated in Figure 6. We adopt the projection of a space curve into planes resulting in knotoid diagrams, to a projection for a bonded protein chain. More precisely, we choose a projection direction determined by two parallel (infinite) lines passing through the termini of the chain and we project the protein chain into the plane that is orthogonal to these lines. We only consider projection directions that give a generic diagram, in the sense that we have only finitely many self-crossing points and that a bonding site is represented in the projection in a parallel or in anti-parallel fashion. The information of each bond is represented with dotted segments connecting the two ends involved. Endowing each self-intersection point with the weaving information of the chain in the space, we obtain an open-ended knotted diagram in the projection plane with the extra information of bonds. We call such a diagram a *bonded knotoid diagram*.

We consider each bonding site in a bonded knotoid diagram locally as a rigid planar formation. As such, a bonding arc in a diagram is not subjected to any topological deformations in the plane such as bending, shrinking or enlarging, and any twisting of the bonding arc is avoided. On the other hand, local strands of bonding sites are topologically flexible. More precisely, we allow on bonded knotoid diagrams the usual Reidemeister moves for knotoids away from the bonds and away from any of the endpoints, and also we allow the bonded moves illustrated in Figure 6, each of which is realized in bonding sites. As seen in Figure 6a–d, the first two moves, namely bonded twist moves 1 and 2, introduce a twisting in the strands neighboring the bonds. These moves result from a 180-degree turn of the bond, about the vertical and the horizontal axes, respectively. The bonded Reidemeister III move allows an edge of the diagram to slide over or under a bond as a whole without any other change in the bonded knotoid diagram. An edge may be located over or under a bonding arc. The bonded slide moves illustrated in Figure 6e,f allow the movement of such an edge located in between the local strands of the bonding site, so that the bonding site is free from any edges other than the bonding arc. The above moves generate an isotopy relation for bonded knotoid diagrams and an isotopy class of bonded knotoid diagrams is a *bonded knotoid*. The isotopy moves of bonded knotoid diagrams are analogous to what is known in graph theory as rigid vertex isotopy moves [27], if one replaces a bonding site with a rigid vertex.

**Figure 7:**
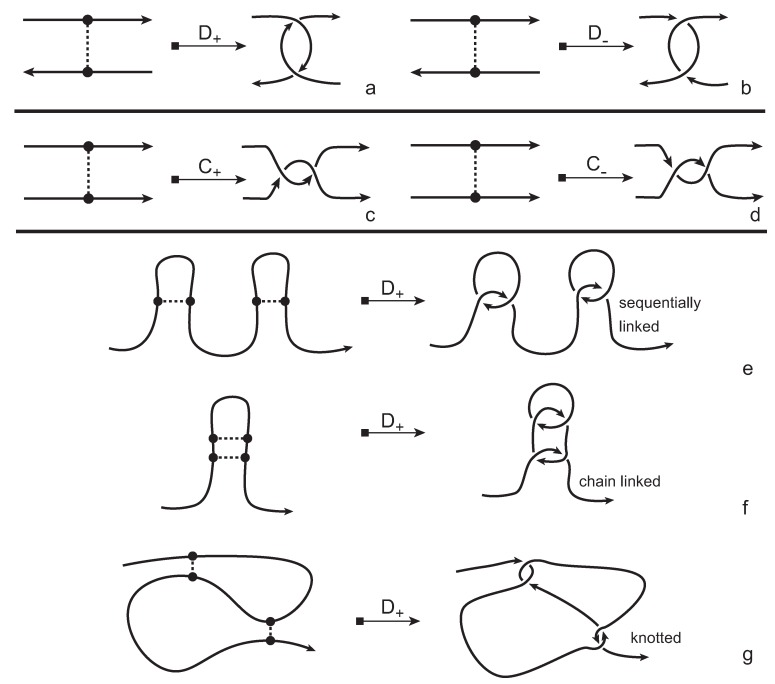
Type *D* (**a**,**b**), and type *C* (**c**,**d**) substitutions. Type *D* substitutions distinguish (**e**) sequential, (**f**) nested and (**g**) pseudoknot-like bonds by applying to the substitution a polynomial invariant for knotoids like the Turaev loop bracket polynomial or the arrow polynomial.

In order to obtain a (multi-) knotoid diagram from a bonded knotoid diagram, we substitute each bonding site by a chosen full twist (a 360-degree twist) using the following convention. If the local strands are directed anti-parallel then we substitute the bonding site by a full twist of the strands along the bonding arc, as illustrated in Figure 7a,b, and the substitution is known as being of type *D*. If the local strands are directed parallel then we substitute the site by a full twist of the strands, as shown in Figure 7c,d and the substitution is known to be of type *C*. Note that insertions of type *D* make disconnections in the diagram, while those of type *C* retain connectivity. Either type of full twists can be positive (right-handed) or negative (left-handed). In this paper, all full-twist substitutions are of a positive type. After replacing all bonding sites we end up with a planar (multi-) knotoid diagram. Besides, the isotopy moves defined on bonded knotoid diagrams are consistent with the isotopy moves defined on knotoid diagrams after making the twist substitutions. It follows that if two bonded knotoid diagrams are isotopic then the corresponding (multi-) knotoid diagrams obtained by full-twist substitutions are isotopic. This means that any topological invariant of knotoids can be used for analyzing the topological type of a knotted bonded open protein chain modeled by a bonded knotoid. There are mainly three types of protein bonds: sequential, nested, and pseudoknot-like type bonds, as illustrated in Figure 7e–g. As we see in the figure, all these types of bonds are detected by type *D* substitutions as compared to the same formations with the bonds ignored. This fact can be proved by applying knotoid invariants such as the Turaev loop bracket polynomial and the arrow polynomial.

**Figure 8:**
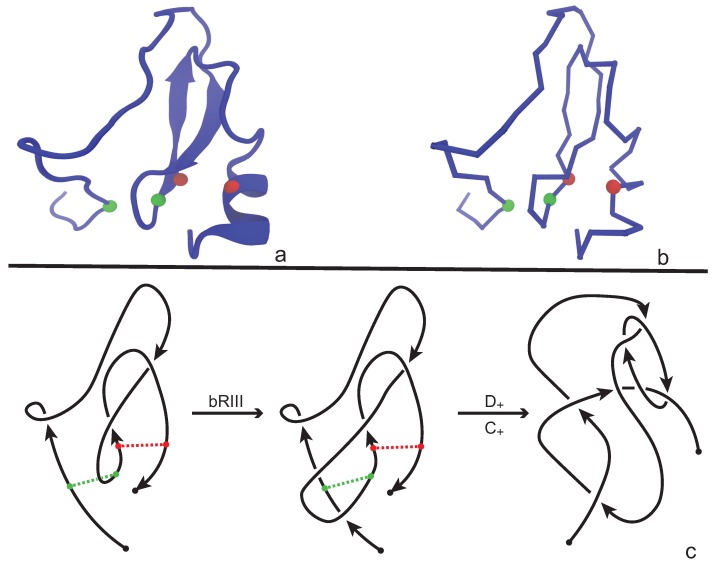
The protein 2LFK as (**a**) a cartoon; and (**b**) a polygonal curve. Same colored spheres indicate that there is a bond between them. More precisely, the green dots correspond to the pair of residues with indices 24 and 51, while the red to the pair 52 and 69. Below (**c**) is the corresponding bonded knotoid diagram and the appropriate substitutions. In the diagram the bonds are represented by colored dashed lines.

An application of our model is illustrated in Figure 8 where we consider the protein with PDB entry 2LFK (NMR solution structure of native TdPI-short) [28]. This protein contains two cysteine bridges that appear between residues 24 and 51 (shown as green beads), and between 52 and 69 (shown as red beads) in Figure 8a,b. For demonstrative reasons we will discuss the application of our model on a single fixed projection, however one has to have in mind that, following Section 3.1, several projections of the backbone have to be analyzed in order to obtain an accurate overview of the topology of the chain. We proceed now and consider the projection of the protein chain that is shown in Figure 8c, which is a bonded knotoid diagram with two bonding sites. Notice that in the green bonding site an arc of the diagram crosses over the bonding arc and so an immediate application of a full-twist is not possible at this state. However, an application of a bonded Reidemeister III move pushes the arc to the left, allowing now the application of a type C+ full-twist since the green bonding site is in a parallel fashion. The situation for the red bonding site is straightforward. Here, we observe that the bonding site is in anti-parallel fashion and so a type D+ full-twist substitution is immediately applied giving rise to a multi-knotoid diagram with two components that can be evaluated with any invariant for knotoids such as the Turaev loop bracket polynomial or the arrow polynomial. Of course, if one drops the bonding information, the structure becomes unknotted. We note here that the same protein has been analyzed in [11,12] for the existence of links where the cysteine bridges are closed with a direct line instead of a full-twist substitution. This explains the difference in the detected link type.

#### An Application to Complex Lassos

**Figure 9:**
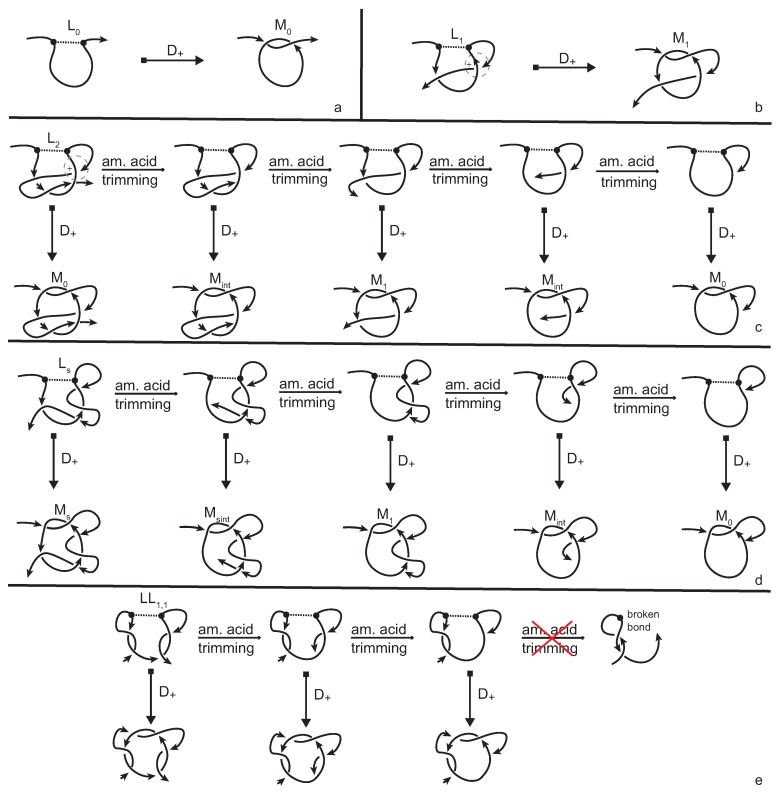
Distinguishing the lassos (**a**) $L_{0}$; (**b**) $L_{1}$; (**c**) $L_{2}$; (**d**) $LL_{1,1}$; and (**e**) $L_{s}$. The crossing the determines the first piercing of the loop is positive and so a type D+ insertion is performed on the lassos. The slipknot in $L_{2}$ is detected through progressive trimming of the C terminus of the three-dimensional curve and evaluating each resulting diagram, while the first two lassos are distinguished immediately by the insertion. Insertions in lassos (**d**,**e**) distinguish them from the types that were already discussed, but not from one another. Trimming the C terminus of the 3D chain leads to different substructures allowing, thus, their distinction.

A lasso is formed when a disulphide bond between two amino acids of the protein backbone creates a closed loop that is called a *cysteine* or *covalent loop* (see Figure 9). The parts of the chain that are not involved in the spanning of the loop are the *tails* of the lasso and if they are short enough, the resulting structure is unknotted. However, in general cases at least one of the tails is long enough to pass through the loop. There is great interest in understanding the impact of the existence of lassos on the function and stability of proteins, as well as the way these structures fold and so it is important to be able to distinguish between different types of lassos. In other words, we would like to know if and how many times a tail is threaded through the loop. Until now, such an analysis was achieved using a technique called minimal surface analysis. This technique is geometrical and it determines how many times a tail pierces the minimal surface that can be spanned by the covalent loop. In what follows, we propose an approach that utilizes bonded knotoid diagrams and we apply it on all motifs that are presented in [9].

The protein chain together with the bonding information, that is the indices of the residues that form the covalent bond, is placed inside a large enough sphere and then is projected on a number of different planes. In this way, a bonded knotoids diagram is obtained from each projection and so we proceed with applying the method that was discussed in Section 3.2. Since the bonding site of every lasso is in an antiparallel fashion, we apply a single positive or negative type *D* full-twist and then we evaluate the resulting diagram. The sign of the full-twist substitution is determined by the sign of the crossing of the diagram that determines the first piercing of the loop.

The simplest lassos are $L_{0}$ and $L_{1}$ in which the tail either does not pierce the loop at all, or does so only once. They are distinguished immediately by our method, since the twist insertion at the site of the cysteine bond produces the non-equivalent multi-knotoids $M_{0}$ and $M_{1}$, respectively (see Figure 9a,b). The cases of lassos $L_{i}$, where i > 1 is the number of times that the tail is threaded through the loop, require more attention. The amount of times that a tail pierces the loop creates slipknot-like forms in the conformation that remain undetected by regular topological tools (knot invariants). Indeed, twist insertion will produce a multi-knotoid diagram that is topologically equivalent to either $M_{0}$ or $M_{1}$, depending on whether *i* is even or odd. Therefore, in order to distinguish lassos of this type, we have to go back to the 3D chain and study all possible substructures of it that include the bonding site. Through progressive trimming of the C terminus of the chain, by projecting, by twist insertions, and by evaluations of the resulting diagrams we can see that for the case of $L_{2}$ (see Figure 9c), the trimming process causes the resulting multi-knotoids to shift between types $M_{0}$, $M_{int}$, and $M_{1}$. During this process, the multi-knotoid of type $M_{int}$, which corresponds to the subchain which has one endpoint inside the loop, is achieved twice which confirms the fact that one tail of the lasso is weaved through the loop twice. The cases of $L_{i}$ with larger number of piercings are totally analogous with the multi-knotoid $M_{int}$ appearing *i* times. Finally, the cases of lassos $LL_{1,1}$ (Figure 9d) and $L_{s}$ (Figure 9e) are even more complicated, since the twist insertion produces the same diagram for both. However, the trimming the C terminus of the 3D chain of $LL_{1,1}$ soon comes to an end. Otherwise, the bonding site will be destroyed, while for the case of $L_{s}$ there is no such issue and thus the chain includes a wider spectrum of non-trivial substructures.

The evaluation of the topological type of such multi-knotoid diagrams can be achieved by the Turaev loop bracket polynomial. Alternatively, one may use the arrow polynomial in order to track the placement of the endpoints of the backbone after each trimming. More precisely, the arrow polynomial gives an estimation for the distance between the endpoints of a diagram, that is the least number of arcs that one has to cross in order to connect the two endpoints. This distance is defined as the *height* of the knotoid [16,17].

## 4. Discussion

In this paper we first refined our methods for studying generic protein chains without an internal connection [4] and then we added an extra layer to the complexity of such analysis by considering protein chains with internal connections, including lassos. For this reason, we introduced a second, robust topological model, which is an adaptation of the topological analysis of RNA chains [13] to the case of proteins that contain disulphide bridges in their conformation. If the bonding information is omitted from the second model, we then revert to the model for generic protein chains. Our methods may provide new insight to the study of knotted proteins since the geometry of the chain is preserved, and also because the information of various bonds can be now taken into consideration. Contrary to the case of [13], our model for bonded proteins considers individual bonds between amino acids instead of contracting stem regions to a single vertex. Additionally, we consider the projections of the 3D chains as possibly bonded, open diagrams (knotoids) instead of closed (knots) [13]. One possible limitation of our models is that the resolution of the results depends on the number projections, where each projection requires a separate computation. This could be computationally demanding, especially for larger proteins. Finally, our topological models are not restricted solely to the study of proteins and so they can be applied to the study of other biomolecules, synthetic polymers, lattice random walks, random open polygons, and others.

## 5. Materials and Methods

### 5.1. Construction of Projection Globes and Maps

The projection map for each protein is created as follows: First the protein structure is imported from the Protein Database (PDB) [29]. It is converted to xyz-coordinates and its backbone is extracted (namely, the coordinates of the Cα atoms). Once the protein chain is reconstructed, it is placed inside a large enough sphere and it is projected to 100 different fixed directions. We consider proteins that do not contain gaps in their conformation. The projections correspond to vectors from the origin of the sphere that point towards vertices uniformly distributed on the surface of the enclosing sphere. Next, the Voronoi cells that correspond to these vertices are computed and each knotoid type is weighted by the area of the associated cell [30]. In order to perform all the above procedures (simplifications of the protein chain, projections and polynomial evalutations of the resulting diagrams) we have used the program Knoto-ID that was developed by Dimos Goundaroulis and Julien Dorier.

### 5.2. The Turaev Loop Bracket

**Figure 10:**
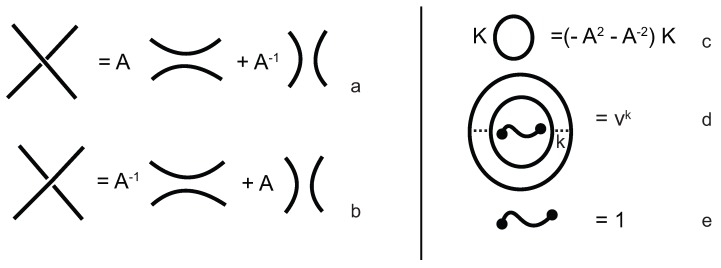
The Turaev loop bracket rules. (**a**,**b**) The smoothing rules; (**c**) the loop value; (**d**) the value for nested loops enclosing a long segment; (**e**) the long segment value.

The Turaev loop bracket polynomial ${\hat{f}}_{K}\left(A,v\right)\in Z\left[A^{\pm 1},v\right]$, is a 2-variable generalization of the Kauffman bracket for planar knotoids, with the added extra variable assigned to nested loop components enclosing a long segment component. The loop bracket polynomial can be also seen as a special case of the three-variable Laurent polynomial $f_{K}\left(A,v,u\right)$ in $Z[A^{\pm 1},v,u^{\pm 1}]$, which is called the extended bracket polynomial when u = 1 [16]. The loop bracket can be defined as follows:

(1) $${\hat{f}}_{K}\left(A,v\right)={\left(-A^{3}\right)}^{-\mathrm{wr}(K)}\langle \langle K\rangle \rangle ,$$

where wr(K) is the writhe of *K*, and 〈〈K〉〉 is the *Turaev loop bracket polynomial*. It is defined by the four rules that are illustrated in Figure 10. The diagrams involved in Figure 10a,b are identical except at one crossing. Further, this rule provides us a recipe to smooth all crossings of the knotoid diagram. At the end of the smoothing process we will obtain a collection of circles together with a single long segment. The rule in Figure 10c means that whenever we have a disjointed circle in a state, we can remove it and multiply by $\left(-A^{2}-A^{-2}\right)$. The rule in Figure 10d assigns the value $v^{k}$ to *k* copies of a circle that contain the long segment, k > 0.

### 5.3. The Arrow Polynomial

**Figure 11:**
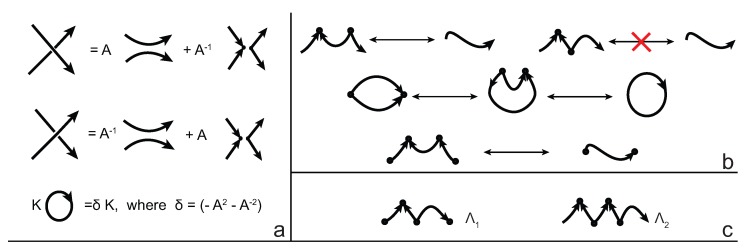
(**a**) Oriented state expansion; (**b**) Reduction rules for the arrow polynomial; and (**c**) a component with one ($\Lambda_{1}$) and two zig-zags ($\Lambda_{2}$), respectively.

The arrow polynomial of knotoids was first introduced in [31] and then applied to knotoids in [17] is a Laurent polynomial with infinitely many commuting variables; $A,\Lambda_{0},\Lambda_{1},\Lambda_{2},\cdots ,\Lambda_{n},\cdots$ The construction of the arrow polynomial is based on the oriented state expansion of the Kauffman bracket, given in Figure 11a. Each crossing of a knotoid diagram is smoothed first in an oriented and then in an disoriented way. A new combinatorial structure arises in the form of a pair of cusps by each disoriented smoothing.

The circular and open components resulting from each type of smoothing of crossings are subjected to the reduction rules given in Figure 11b, each of which arises from the topological invariance in the plane. We keep the information of cusps appearing in states by not eliminating a state component with cusps forming zig-zags but rather assigning a new variable to this type of components. On the other hand, cusps pointing to the same local side in the plane are eliminated. Eventually, each circular component of a state contributes to the polynomial as a factor $\left(-A^{2}-A^{-2}\right)$ for that state. Additionally, each open state component with *i* zig-zags is assigned the variable $\Lambda_{i}$ . We define the normalized arrow polynomial as the following sum over all states *S* of a knotoid diagram *K*, where we denote the number of components in *S* by ∥S∥, and $\Lambda_{i}$ is the variable assigned to the long component of *S*.

(2) $$A\left[K\right]={\left(-A^{3}\right)}^{-\mathrm{wr}(K)}\;\sum_{S}A^{\left(⋕\;\mathrm{of}\;A\;\mathrm{smoothings})-(⋕\;\mathrm{of}\;B\;\mathrm{smoothings}\right)}{\left(-A^{2}-A^{-2}\right)}^{∥S∥-1}\Lambda_{i}.$$

**Figure 12:**
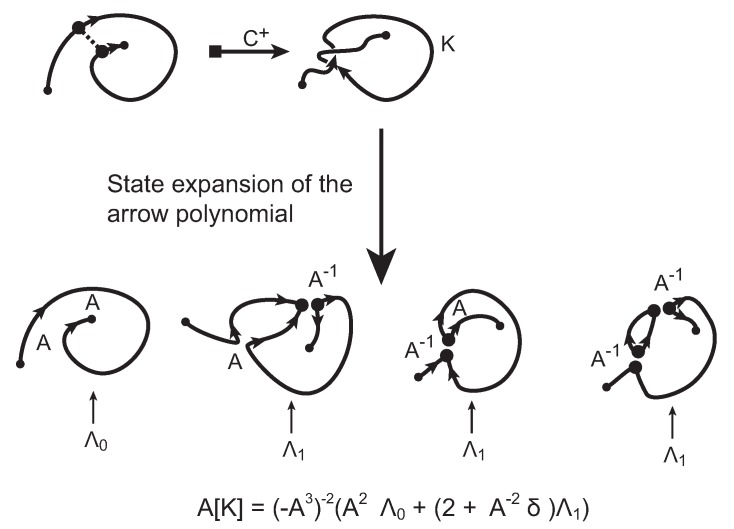
Computing the arrow polynomial of a simple example.

Additional variables assigned to long state components with irreducible cusps (in zig-zag form) make the arrow polynomial a strong invariant for understanding the height of a knotoid. As shown in [17], the maximum Λ-degree of the arrow polynomial, that is, the maximum integer *i* that we see as an index of $\Lambda_{i}$-variables in the arrow polynomials, provides a lower bound for the height of a knotoid. See Figure 12 for an example of a calculation of an arrow polynomial obtained from an insertion to a simple bonded knotoid.

We note here that the arrow polynomial can be generalized to a loop arrow polynomial for planar knotoids, in analogy with the Turaev loop bracket polynomial, by assigning different variables to circular state components in a state depending on whether they enclose a long segment (with or without irreducible cusps, respectively) or not. This polynomial will be a subject of a subsequent paper.

## Supplementary Materials

The following are available online at www.mdpi.com/2073-4360/9/9/444/s1, Video S1: Line isotopy, Video S2: Change of projection plane.

## Abbreviations

The following abbreviations are used in this manuscript:

KMT algorithm	Koniaris, Muthukumar, Taylor algorithm
PDB	Protein Database
